# A Comparison of the In Vitro Effects of 2’Fucosyllactose and Lactose on the Composition and Activity of Gut Microbiota from Infants and Toddlers

**DOI:** 10.3390/nu13030726

**Published:** 2021-02-25

**Authors:** Pieter Van den Abbeele, Norbert Sprenger, Jonas Ghyselinck, Benoît Marsaux, Massimo Marzorati, Florence Rochat

**Affiliations:** 1 ProDigest, Technologiepark 82, 9052 Zwijnaarde, Belgium; pieter.vandenabbeele@telenet.be (P.V.d.A.); Jonas.Ghyselinck@prodigest.eu (J.G.); Benoit.Marsaux@prodigest.eu (B.M.); 2 Nestlé Institute of Health Sciences, Société des Produits Nestlé S.A., Vers-Chez-Les-Blanc, CH-1000 Lausanne, Switzerland; norbert.sprenger@rdls.nestle.com; 3 Center for Microbial Ecology and Technology (CMET), Faculty of Bioscience Engineering, Ghent University, Coupure Links 653, 9000 Ghent, Belgium

**Keywords:** 2’fucosyllactose, human milk oligosaccharides, infant gut microbiota, lactose, mucosal simulator of the human intestinal microbial ecosystem, prebiotic, toddler gut microbiota

## Abstract

Because of the recognized health benefits of breast milk, it is recommended as the sole nutrition source during the first 6 months of life. Among the bioactive components are human milk oligosaccharides (HMOs) that exert part of their activity via the gut microbiota. Here, we investigated the gut microbiota fermentation of HMO 2’fucosyllactose (2’-FL), using two in vitro models (48 h fecal incubations and the long-term mucosal simulator of the human intestinal microbial ecosystem [M-SHIME^®^]) with fecal samples from 3-month-old breastfed (BF) infants as well as 2–3 year old toddlers. The short-term model allowed the screening of five donors for each group and provided supportive data for the M-SHIME^®^ study. A key finding was the strong and immediate increase in the relative abundance of *Bifidobacteriaceae* following 2’-FL fermentation by both the BF infant and toddler microbiota in the M-SHIME^®^. At the metabolic level, while decreasing branched-chain fatty acids, 2’-FL strongly increased acetate production together with increases in the health-related propionate and butyrate whilst gas production only mildly increased. Notably, consistently lower gas production was observed with 2’-FL fermentation as compared to lactose, suggesting that reduced discomfort during the dynamic microbiome establishment in early life may be an advantage along with the bifidogenic effect observed.

## 1. Introduction

Gut microbes play a key role in human health, including the development of the immune system [1,2,3] and host metabolism [4,5]. Gut microbiome fermentation of indigestible dietary glycans into short-chain fatty acids (SCFAs); mostly acetate, propionate, and butyrate, are important in maintaining host health throughout life [6]. In terms of composition, infants have a less diverse gut microbial community with a lower representation of members of *Bacteroidetes*, *Firmicutes,* and *Archaea* compared with adults [7,8]. In contrast, the infant gut microbial community is dominated by members of the *Bifidobacteriaceae* family [7], which are specialized to ferment carbohydrates derived from breast milk into acetate and lactate [9]. This *Bifidobacteriaceae* dominance persists during the first year of life, followed by a decreased relative percentage as the infant ages [7,10]. The gut microbial community continues to change and becomes more diverse and adult-like by age three [7]. With increasing age, cross-feeding bacteria (such as acetate and/or lactate converters) begin to more heavily colonize the gut, resulting in an increased production of propionate and butyrate [11,12]. As the microbial community evolves, members of the *Bacteroidetes* and *Firmicutes* phylum eventually comprise approximately 90% of the gut microbiota [8,13]. Both gas production and pH can fluctuate depending upon the composition of the gut microbial community [14,15]. pH can affect community diversity and the production of SCFAs [14], and therefore the effects of active substances on pH are important to consider. Additionally, gas production can lead to discomfort, so active substances that ultimately result in reduced gas production are desirable. While in vivo studies have provided interesting knowledge and are key to prove the final efficacy of an active agent, they are often rather descriptive and do not allow for the gleaning of insights into the mechanisms by which specific glycans affect the gut microbiota, leaving a clear need for well-controlled in vitro studies to address such questions.

Breastfeeding is related to numerous benefits for the mother and infant [16,17,18]. Several decades of research have shown that breast milk contains a variety of immunomodulatory, anti-inflammatory, and antimicrobial substances that help to reduce short- and long-term morbidity risks and positively influence the child’s cognitive and psychomotor development [19,20,21]. As a result of the numerous benefits of breastfeeding, the World Health Organization (WHO) recommends exclusive breastfeeding up to 6 months of age, with continued breastfeeding along with appropriate complementary foods up to two years of age and beyond [22,23].

Among the many bioactives found in human breast milk is a large amount and diversity of carbohydrates, namely the milk sugar lactose, and elongations of lactose by the building blocks fucose, sialic acid, lacto-N-biose, and N-acetyl-lactosamine that are generally summarized as human milk oligosaccharides (HMOs) [24]. Concluded from basic research, HMOs are expected to act via different routes including the support of growth and metabolic activity of specific gut bacterial taxa such as specific bifidobacteria [25]. Among the HMOs shown to modulate specific bifidobacteria is 2’fucosyllactose (2’-FL) [26,27,28]. 2’-FL is a trisaccharide composed of L-fucose, D-galactose, and D-glucose units. Although >150 different HMOs are described, the 20-30 HMOs that are generally quantified in different studies likely correspond to the vast majority by mass, and 2’FL is generally the most abundant HMO in breast milk [29,30]. It is one of the HMOs available today in large quantities from synthetic production and it is shown to be safe and beneficial for infant nutrition [31,32,33,34,35,36]. Studies have shown that the addition of either 2’-FL alone or 2’-FL in combination with Lacto-N-neotetraose (LNnT; another HMO) to infant formula supports immune and gut health [31,35,37]. 2’-FL is a trisaccharide composed of L-fucose, D-galactose, and D-glucose units. While 2’-FL has shown potential for improving the health of formula-fed infants, little is known about the mechanisms by which these improvements are accomplished. Further, beyond the first few months of age there is currently a lack of knowledge on possible roles of 2’-FL or other HMOs for toddlers who may still get HMOs if breastfeeding is continued. To what extent recent observations of HMO supplementation in adults can inform possible roles in toddlers has to be evaluated experimentally [38,39].

The gut microbiota composition varies largely among individuals [7,40]. This is the case not only for adults, but also for infants and toddlers [7,41,42]. In fact, variation between human individuals is reported to be greater among children (0–3 years of age) than adults [7]. This can be explained by a multitude of factors such as vaginal versus cesarean section delivery, breast milk versus formula feeding, or the use of antibiotics, all factors that can impact the early colonization and subsequent development of the gut ecosystem [43]. As these factors result in different microbial community composition, they can thus also affect the utilization of dietary glycans [44,45]. Careful consideration of these inter-individual differences among infants is thus needed when studying the effects of dietary glycans on their gut microbiome.

Therefore, the current study was conducted to address the lack of understanding of how 2’-FL affects the gut microbiome in infants and toddlers, while taking interindividual differences in gut microbiota composition into consideration. In the first part, we applied our recently developed short-term in vitro fermentation model [46] to study the effect of a single dose of 2’-FL versus lactose on gut microbiota from five breastfed (BF) infants (3 months old) and five toddlers (2 years old). The assessment involved parameters related to microbial activity and *Bifidobacterium* levels. This donor screening allowed for the selection of one representative donor from each age group for the second part of the study that considered a more in-depth investigation of the effects of repeated intake of 2’-FL and lactose over a period of 3 weeks on the gut microbiota using the mucosal simulator of the human intestinal microbial ecosystem (M-SHIME^®^). This model simulates both longitudinal differences (proximal versus distal colon) as well as differences between luminal and mucosal microbiota [47]. Besides assessing microbial activity, microbial community composition was assessed via 16S-targeted Illumina sequencing.

## 2. Materials and Methods

### 2.1. Test Products

All chemicals were obtained from Merck (Darmstadt, Germany) unless stated otherwise. 2’-FL used in this study was provided by Glycom A/S (Horsholm, Denmark; purity > 94% (*w/w*)), while lactose was acquired from Oxoid (Aalst, Belgium).

### 2.2. Experimental Design of Short-Term Incubations (Part 1) and Long-Term M-SHIME^®^ Study (Part 2)

**Figure 1:**
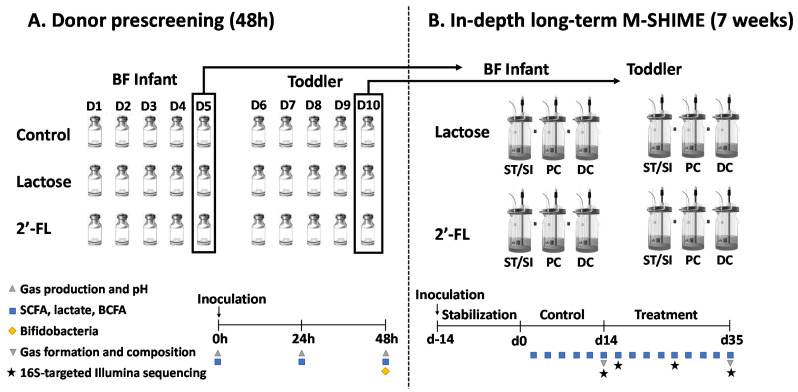
Schematic representation of the experimental set up: (**A**) Short-term incubations (part 1) to screen fecal samples from five BF infant and five toddler donors (48 h); (**B**) Long-term M-SHIME^®^ study (part 2) using the selected fecal samples from the screening of a single BF infant and toddler donor, respectively (7 weeks). 2’-FL = 2’fucosyllactose; BF = breastfed; SFCA = short-chain fatty acid; BCFA = branched-chain fatty acid; ST/SI = stomach/small intestine; PC = proximal colon; DC = distal colon; M-SHIME^®^ = mucosal simulator of the human intestinal microbial ecosystem.

The first part of the study considered short-term fecal batch incubations of a single dose (5 g/L) of 2’-FL and lactose versus a blank control, when administered to a fecal microbiota from five different BF infants (3 months old; D1–5) and five toddlers (2–3 years old; D6-10), resulting in 30 independent reactors (Figure 1A). The incubation approach was identical to the one recently described [46], with the following modifications: colonic background medium (K_2_HPO_4_ 4.8 g/L; KH_2_PO_4_ 14.9 g/L; NaHCO_3_ 2.0 g/L; yeast extract 2.0 g/L; peptone 2.0 g/L; mucin 1.0 g/L; cysteine 0.5 g/L; polyoxyethylene (20) sorbitan monooleate 2.0 mL/L), and anaerobic phosphate buffer (K_2_HPO_4_ 8.8 g/L; KH_2_PO_4_ 6.8 g/L; sodium thioglycolate 0.1 g/L; sodium dithionite 0.015 g/L). Samples were collected at 0 h, 24 h, and 48 h for microbial metabolic activity analysis: pH, gas production, SCFA, lactate, and branched-chain fatty acid (BCFA) production. At 48 h, *Bifidobacterium* levels were quantified via qPCR.

The second part of the study considered assessing the effects of repeated intake of 2’-FL and lactose on the gut microbiota using the M-SHIME^®^ (ProDigest and Ghent University, Ghent, Belgium) (Figure 1B).

The configuration of the model parameters were adapted from the model for human adults as described by Van den Abbeele et al. (2019) [47] to represent the gastrointestinal tract of 3-month-old infants, as reported in De Boever et al. (2001) [48], with additional minor adaptations for the simulation of approximately 2–3 year old toddlers as specified below. This semi-continuous model allowed for the parallel investigation of four different arms consisting of the two age groups (BF infant and toddler) and two treatments (2’-FL and lactose) in a single setup consisting of 12 reactors (so-called QUAD-M-SHIME).Each arm consisted of a first reactor that simulated over time the stomach and small intestine and that operated according to a fill-and-draw principle, with peristaltic pumps adding a defined amount of nutritional medium (140 mL) to the stomach at pH 3 and after 1.5 h incubation followed by addition of pancreatic and bile liquid (60 mL) in the small intestine (pH 6). After another 1.5 h incubation in the small intestine, the intestinal suspension was pumped to the second and third reactors that simulated the proximal colon (PC) and distal colon (DC). These reactors were continuously stirred with constant volume (300 mL in PC and 500 mL in DC) and pH control (pH 5.8–6.0 in PC and pH 6.0–6.5 in DC), resulting in a total colonic retention time of 32 h. Each colonic reactor included a simulation of the mucosal environment by the inclusion of mucin-coated microcosms as described by Van den Abbeele et al. (2013) [49]. Reactor feed composition for the simulation of BF infants consisted of 4 g/L mucin, 1 g/L yeast extract, 0.2 g/L cysteine, and around 10 g/L digested milk fragments (4.8 g/L lactose, 0.5 g/L casein, and 4.6 g/L lactalbumine). For the simulation of a toddler microbiota, the digested milk fragments were decreased by half and replaced by carbohydrates to simulate the intake of solid food, i.e., 2 g/L starch, 1 g/L pectin, 1 g/L glucose, 1 g/L cellobiose. For both age groups, the pancreatic and bile liquid consisted of 2.5 g/L NaHCO3, 0.9 g/L pancreatin and 4 g/L oxgall. The fecal inoculum was prepared as described above with 20% (*w*/*v*) as compared to part 1. After inoculating 5% (*v*/*v*) in the colonic reactors, the experiment was initiated. The SHIME^®^ cabinet and integrated software were run according to the manufacturer’s instructions (ProDigest, Ghent, Belgium). The experimental timeline of the M-SHIME run consisted of a two-week stabilization period (d-14 to d0), during which the fecal microbiota differentiated to communities representative for a specific colon region, followed by a two-week baseline period (d0 to d14) and a three-week treatment period (d14 to d35).

During the treatment period, 2’-FL and lactose were administered at 10 g/L in the nutritional medium resulting in a concentration of 7 g/L in the small intestinal suspension that enters the proximal colon. Gas production and composition were assessed during the M-SHIME^®^ experiment using an offline setup that involved collecting samples from the proximal colon and subsequently treating them with lactose or 2’-FL in closed reactors for 48 h. This incubation strategy was identical to the one in the first part of the study (short-term incubations) except for the inoculum that was derived from the M-SHIME^®^ model (7 mL), while in the first part of the study, it involved adding 1 mL fecal slurry. The offline gas formation assessment was performed on samples collected before (d14) and after treatment (d35) to assess whether repeated administration of 2’-FL or lactose in the M-SHIME^®^ resulted in altered gas production by the microbiota.

Mucosal and luminal samples were obtained at the same time point in each reactor for microbiota analysis.

### 2.3. Microbial Community Analysis by qPCR

Samples collected after 48 h during the short-term incubations were evaluated for the total amount of *Bifidobacterium* species by qPCR. DNA was isolated as described before [50] with minor modifications [51] from either 1 mL luminal samples or 0.1 g mucus samples. Subsequently, qPCR was performed using a QuantStudio 5 Real-Time PCR system (Applied Biosystems, Foster City, CA, USA). Each sample was run in technical triplicate and outliers with more than 1 C_T_ difference were omitted. The qPCRs were performed as described previously with the primers Bif243F (5′-TCGCGTCYGGTGTGAAAG-3′) and Bif243R (5′CCACATCCAGCRTCCAC-3′) [52]. Results are reported as log(16S rRNA gene copies/mL).

### 2.4. Microbial Community Analysis by 16S rRNA Gene Sequencing

Microbial community composition was assessed before (d14) and after treatment with 2’-FL and lactose (d16, d26, and d35). Samples were sent out to LGC Genomics GmbH (Berlin, Germany) for next-generation 16S rRNA gene amplicon sequencing of the V3–V4 region. Library preparation and sequencing were performed using an Illumina MiSeq platform with v3 chemistry. The 341F (5′-CCTACGGGNGGCWGCAG-3′) and 785R (5′-GACTACHVGGGTATCTAAKCC-3′) primers were used as previously described [53], with the reverse primer being adapted to increase coverage. Quality control PCR was conducted using Taq DNA Polymerase with the Fermentas PCR Kit according to the manufacturers’ instructions (Thermo Fisher Scientific, Waltham, USA). The DNA quality was verified by electrophoresis on a 2% (*w*/*v*) agarose gel for 30 min at 100 V. Bioinformatics analysis of amplicon data was performed as previously [54]. Briefly, The mothur software package (v.1.33.3) and guidelines were used to process the amplicon data generated by LGC Genomics. In short, after assembling forward and reverse reads, contigs with a length between 441 and 467 bases were aligned to the mothur formatted silva_seed release 119 alignment database, trimmed between positions 6388 and 25316, to be compatible with the 341F/785R primers [55]. After removing non-aligning sequences as well as sequences containing homopolymer stretches of more than 12 bases, sequences were pre-clustered, allowing up to 4 differences. UCHIME was applied to remove chimera [56]. Subsequently, sequences were classified, by means of a naive Bayesian classifier, against the RDP 16S rRNA gene training set, version 14, with an 80% cut-off for the pseudobootstrap confidence score. All sequences that were classified as Eukaryota, Archaea, Chloroplasts, and Mitochondria were removed and only bacterial sequences were retained. Also, if sequences could not be classified at all (even at (super)Kingdom level) they were removed. Sequences were binned into Operational Taxonomic Units (OTU’s) within each order identified by the preceding classification step. An OTU is defined in this manuscript as a collection of sequences with a length between 402 and 427 nucleotides that are found to be more than 97% similar to one another in the V3-V4 region of their 16S rRNA gene after applying Opticlust clustering [57,58,59,60]. Taxonomy was assigned using the RDP version 16 and silva.nr_v123 database [56,57,58]. The shared file, containing the number of reads observed for each OTU in each sample, was loaded into Microsoft^®^ Excel^®^ 2016 MSO (16.0.11901.20070) (Redmond, USA). Reads occurring only 5 times in all samples were removed, as they were supposedly artefacts or bacteria that were not having any biological impact. For the most abundant OTUs, the sequences retrieved from 3% dissimilarity level fasta file obtained in mothur were classified through the RDP web interface using the RDP SeqMatch tool. The database search was restricted to type strains with only near-full-length and good quality sequences. The sequences were blasted in NCBI against the 16S rRNA gene sequences, selecting only type material, with optimization of the BLAST algorithm for highly similar sequences (accession date: December 2018) [56,58,59]. Although identification to the species level based on short 300 bp reads may involve some ambiguity, the most likely species classification of a few interesting OTUs is reported in the results sections. In the event of inconsistencies in the results of the RDP SeqMatch tool and NCBI BLAST, no species level classification is provided. The results are presented as proportional values. Diversity indices (Shannon, Chao1), evenness, and richness were calculated using Calypso software version 8.72 [61].

### 2.5. Metabolic Analysis

pH measurements were performed using a Senseline pH meter F410 (ProSense, Oosterhout, The Netherlands). Gas formation was measured using a pressure meter to which a needle was connected (hand-held pressure indicator CPH6200; Wika, Echt, The Netherlands). Gas-phase composition was analyzed using a compact GC (Global Analyser Solutions, Breda, The Netherlands), equipped with a Molsieve 5A pre-column and Porabond column (for CH4, O2, H2, N2), a Rt-Q-bond pre-column, and column (for CO2, N2O, and H2S), and a thermal conductivity detector. The parameters used to evaluate the activity of the gut microbiota were monitored 3x/week during the baseline (d3/5/7/10/12/14) and treatment period (d16/19/21/23/26/28/30/33/35). SCFA (acetate, propionate, and butyrate) and BCFA (isobutyrate, isovalerate, and isocaproate) were determined as described previously [62]. Lactate production was assessed with a kit (R-Biopharm, Darmstadt, Germany), according to the manufacturer’s instructions.

### 2.6. Statistical Methods

All statistical analyses were performed using GraphPad Prism version 8.2.0 (435) for Windows (GraphPad Software, San Diego, CA, USA). All formal hypothesis tests were conducted at the 5% significance level (α = 0.05). Comparison of data from the control and treatment conditions on microbial metabolic and composition markers of the five BF and five toddler donors was made by calculating the average per condition and then by performing a two-way ANOVA with Bonferroni correction. The equality of variance and normality of the data and residuals were checked based on visual inspection of homoscedasticity and QQ-plots, respectively. Principal component analysis (PCA) was performed using ClustVis (https://biit.cs.ut.ee/clustvis/ (accessed on 16 October 2020), accessed on the 8th of May, 2020) after standardizing data [63]. Briefly, dimensions were first centered and scaled before being uploaded in ClustVis (Metsalu, T. & Vilo, J., 2015, Nucleic Acids Research), in order to reduce the impact of large units. Principal components were calculated using the SVD method with imputation in pcaMethods [64] R package, which performs imputation and Singular Value Decomposition (SVD) iteratively until estimates of missing values converge. For the data processing, unit variance scaling method was performed using pcaMethods R package, which divides the values by standard deviation so that each row has variance equal to one. Variables were grouped per treatment and sampling time, and shaped depending on the original donor.

For each microbial metabolic and growth marker (pH, gas production, SCFA, BCFA, lactate, and qPCR), the increase or decrease from either 0 h to 24 h or from 0 h to 48 h of incubation was used to create a joint PCA biplot that allowed comparing the five BF infants’ or the five toddler’s microbial changes induced by lactose or 2’-FL during the first part of the study. Comparison of the data of the baseline, lactose and 2’-FL conditions on microbial metabolic and composition markers of the two selected donors was made by performing a two-way ANOVA with Bonferroni correction. The approximation of the normal distribution of the data was assumed due to the small sample size [65], and the homoscedasticity across the samples was checked by plotting the residuals, although the sample size was equal.

### 2.7. Ethics

Fecal samples of the five BF infants and five toddlers were collected according to the ethical approval of the University Hospital Ghent (reference number B670201836585). This involved obtaining informed consent of legal representatives for inclusion.

## 3. Results

### 3.1. Effect of 2’-FL versus Lactose on Microbial Activity and Bifidobacterium Levels in Short-Term Incubations

To select representative donors for the long-term M-SHIME^®^ study, five BF infants (D1–D5) and five toddlers (D6–D10) were screened based on their fecal microbiota fermentation of lactose and 2’-FL (versus a blank control) during 48 h incubations.

**Figure 2:**
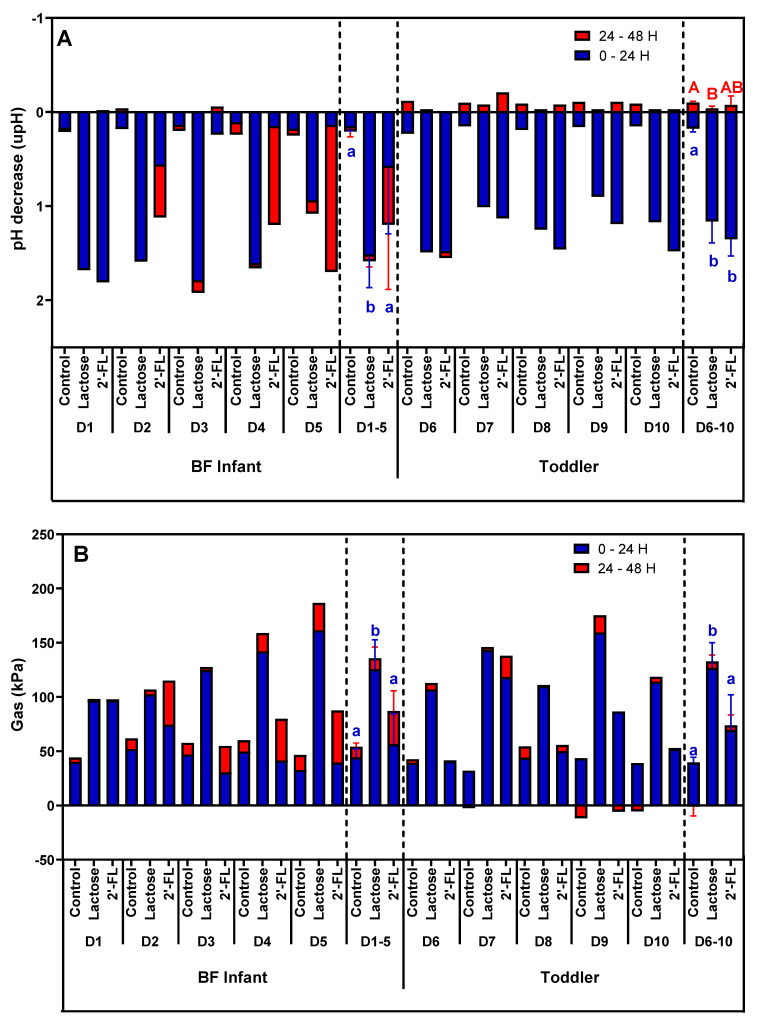
Effect of lactose or 2’-FL on fermentation parameters in short-term colonic incubations. Bars represent changes in (**A**) pH and (**B**) gas formation during two time intervals (0–24 h and 24–48 h) of 48 h fecal batch incubations for five BF infants (D1, D2, D3, D4, and D5) and five toddlers (D6, D7, D8, D9, and D10), upon treatment with lactose or 2’-FL versus a blank control. Besides presenting the data for each donor separately, the average and standard deviation within age group is also presented (D1–5; D6–10). BF = breastfed; 2’-FL = 2’fucosyllactose; D = donor. Significant differences between the average control and the two treatments per age group, as tested with a two-way ANOVA with Bonferroni correction, are indicated with different letters (0-24 h: a, b; 24-48 h: A, B; *p* <0.05).

While pH changes for the control were minimal and similar among the different donors, both lactose and 2’-FL treatment resulted in strong pH decreases (Figure 2A). There was marked interindividual variation among the BF infant donors (D1–D5) in response to 2’-FL treatment, with no (D3) or a delayed acidification between 24–48 h (D2, D4, and D5). As a result, lactose more strongly decreased the pH between 0–24 h compared to 2’-FL for BF infants. After 48 h of incubation, the pH decrease was still more profound for lactose (mostly due to D2, D3, and D4), yet, 2’-FL had also greatly decreased pH compared to the blank at that point. In contrast to the BF infants, there was less interindividual variation in pH changes upon lactose or 2’-FL treatment for the five toddlers (D6–D10), with rapid pH decreases for both substrates within 24 h. As compared to BF infants, 2’-FL decreased pH more strongly, with a greater pH decrease being noted for 2’-FL compared to lactose for each donor, though not reaching statistical significance when averaged over the five donors.

Gas formation was minimal and similar for the control incubations of all donors tested (Figure 2B). Upon 2’-FL treatment, the fecal microbiota of BF infants again displayed considerable interindividual variation with no additional (D3) or a delayed gas formation (D2, D4, and D5). In contrast, lactose resulted in a strong and immediate (within 24 h) increase in gas production for the BF infants. As a result, overall gas production (D1–D5) was significantly higher with lactose than with 2’-FL. Further, there was again less interindividual variation for toddlers (compared to BF infants) and as was observed for BF infants, significantly more gas was produced with lactose versus 2’-FL treatment (Figure 2B).

**Figure 3:**
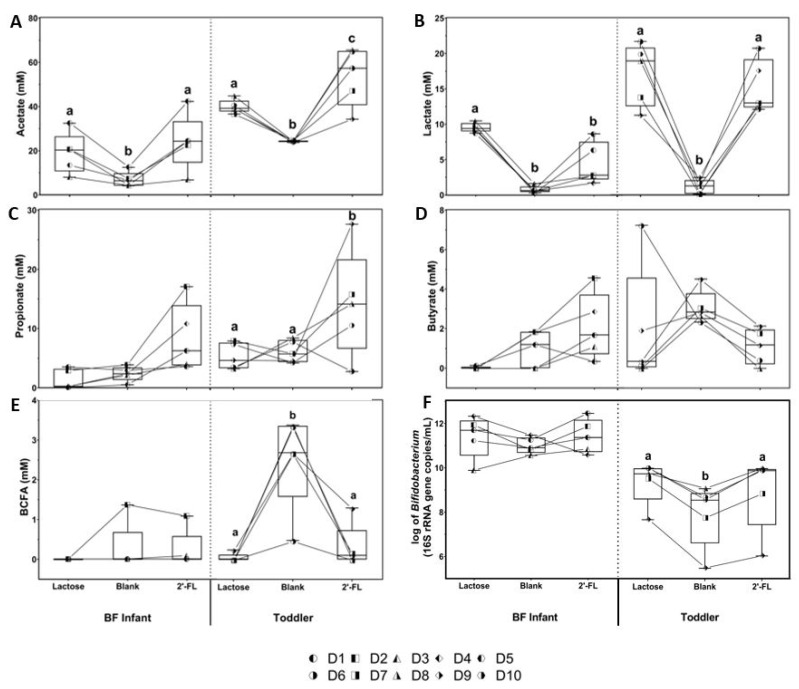
Effect of lactose or 2’-FL on bacterial metabolites and *Bifidobacterium* levels in short-term colonic incubations. Bars represent changes in (**A**) acetate, (**B**) lactate, (**C**) propionate, (**D**) butyrate, (**E**) BCFA, and (**F**) *Bifidobacterium* levels during short-term fecal batch incubations (0–48 h) for five breastfed (BF) infants (D1, D2, D3, D4, and D5) and five toddlers (D6, D7, D8, D9, and D10), upon treatment with lactose or 2’-FL versus a blank control (*n* = 5, 1 each from 5 individual donors). BF = breastfed; 2’-FL = 2’fucosyllactose; D = donor; BCFA = branched-chain fatty acids; SCFA = short-chain fatty acids. Significant differences between the average control and the two treatments per age group, as tested with a two-way ANOVA with Bonferroni correction, are indicated with different letters (a, b, c; *p* <0.05).

Levels of individual SFCAs, lactate, BCFAs, and *Bifidobacterium* (16S rRNA gene copies/mL) are shown in Figure 3. Treatment with either lactose or 2’-FL significantly increased acetate production compared to the blank control (Figure 3A). This acetate increase was observed for both BF infant and toddlers and was most pronounced with 2’-FL treatment in the toddler samples. Lactate production was also higher with lactose and 2’-FL treatment for both BF infant and toddler samples compared to the blank control (Figure 3B), although not reaching statistical significance with 2’-FL treated stool microbiota from BF infants. Lactose increased lactate production consistently in all BF infant samples, while 2’FL did not. Compared to blank controls, both propionate and butyrate production showed a slight increase that did not reach statistical significance with 2’-FL, but not with lactose for BF infant samples. Propionate was significantly higher for toddler samples treated with 2’-FL, but not with lactose, compared to blank controls (Figure 3C). Apparent butyrate production changes by either lactose or 2’-FL treatment for BF infant or toddler samples did not reach statistical significance (Figure 3D). Under all conditions and for all but one BF infant samples, BCFA production was very low. Toddler samples produced the highest BCFA levels in the blank control samples, while significantly lower amounts were produced with both the lactose and 2’-FL treatments (Figure 3E). There was an overall tendency for lactose and 2’-FL treatment to increase the levels of *Bifidobacterium* compared to the blank control that reached significance for the toddler simulation. In the BF infant samples, a relatively high variability in the individual donor responses was observed, with several samples that seemingly did not respond to 2’-FL with increased growth of the *Bifidobacterium* population (Figure 3F). This contrasts the observed acetate response, a typical *Bifidobacterium* metabolite, of almost all BF infant samples to 2’-FL (Figure 3A).

**Figure 4:**
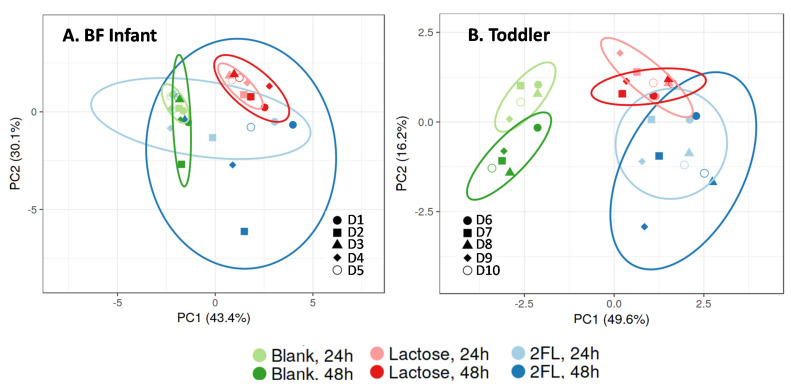
Effect of lactose or 2’-FL on microbial activity and *Bifidobacterium* populations short-term colonic incubations. Principal component analysis (PCA) plots for (**A**) BF infant fecal samples (*n* = 5, 1 each from 5 individual donors) and (**B**) toddler fecal samples (*n* = 5, 1 each from 5 individual donors) representing metabolic data (pH, gas, SCFA, BCFA, and lactate) and *Bifidobacterium* levels obtained for blank control, lactose, or 2’-FL treatments after 24 h and 48 h. Ellipses indicate clusters identified after automatic classification based on PCA scores at 95% confidence interval. BCFA, branched-chain fatty acid; BF = breastfed; 2’-FL = 2’fucosyllactose; D = donor; PC = principal component; SCFA, short-chain fatty acid.

The overall effects of the lactose and 2’-FL treatments on metabolic markers (pH, gas, SCFA, BCFA, and lactate) and *Bifidobacterium* levels (at 24 and 48 h) were visualized in a single plot using PCA (Figure 4). This illustrates that the interindividual differences among donor samples were more pronounced for BF infants than for toddlers. Despite this, there were several consistent findings. First, lactose exerted an immediate treatment effect (within 24 h) that was stable at 48 h for both BF infant and toddler samples. Then, treatment with 2’-FL differed from lactose as the samples clustered in a different region. Moreover, the effect of 2’-FL treatment was more delayed, particularly for BF infant samples. D5 (BF infant) and D10 (toddler) were chosen for a more in-depth M-SHIME^®^ study, as they fell in the middle of the 95% confidence intervals for treatment effects of both lactose and 2’-FL.

### 3.2. Long-Term M-SHIME^®^ Study

#### 3.2.1. Microbial Composition in the M-SHIME^®^ Model Simulating a BF Infant or Toddler Stool Sample

To gain insight into the overall microbial colonization of the M-SHIME^®^ model for both the BF infant and toddler simulation, family-level data was averaged over all study arms and along the entire experiment (d14, d16, d26, and d35), and compared to the composition of the original inocula (Table S1). This revealed that 4–7 weeks after inoculation, almost all families present in the original inocula were still present in the M- SHIME^®^ model, thus confirming that this in vitro model was able to maintain a large part of the bacterial taxa of the inocula. This included the maintenance of the high levels of *Bifidobacteriaceae* that was an abundant group in the inocula of the BF infant and toddler under investigation. Further, the toddler M-SHIME^®^ was colonized by considerable levels of *Bacteroidaceae, Lachnospiraceae*, and *Ruminococcaceae* that were also part of the toddler inoculum. As a remark, *Veillonellaceae* were overrepresented in samples derived from the in vitro model. Finally, significant differences in relative abundance between the proximal and distal colon (indicated in bold in Table S1), and between the luminal and mucosal compartments (indicated in italic in Table S1), highlight intrinsic differences in the microbial communities in each of these four regions. As an example, *Bacteroidaceae* were enriched in the luminal environment of the toddler simulation reaching abundances of around 12% in the lumen and between 3.64–5.11% in mucus.

#### 3.2.2. Effect of Repeated Administration of 2’-FL versus Lactose on Microbial Composition in the M-SHIME^®^

**Figure 5:**
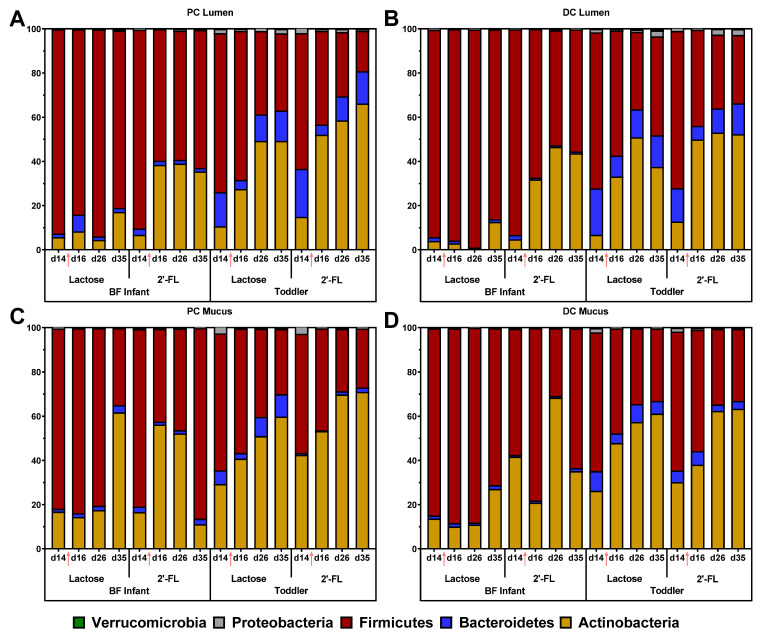
Effect of long-term administration of lactose or 2’-FL on infant and todder microbiota in vitro. Stacked bars represent proportional abundance at the phylum level (%) for BF infant or toddler microbiota before (d14) and after treatment with lactose or 2’-FL (d16, d26, and d35) in the M-SHIME^®^: (**A**) PC lumen, (**B**) DC lumen, (**C**) PC mucus, (**D**) DC mucus. BF = breastfed; 2’-FL = 2’fucosyllactose; d = day; PC = proximal colon; DC = distal colon; M-SHIME^®^ = mucosal simulator of the human intestinal microbial ecosystem. Arrows indicate the start of treatment with lactose or 2’-FL.

The effects of lactose or 2’-FL treatment on the BF infant and toddler microbial community composition at phylum level are shown in Figure 5, while changes at the family level are shown in Table 1 and Table S2–S4 for the lumen of the proximal or distal colon as well as the mucus of the proximal or distal colon. First, in the lumen of the proximal colon 2’-FL immediately increased *Actinobacteria* (from d14 to d16), both for the BF infant and toddler simulations. This was due to a marked stimulation of *Bifidobacteriaceae* by 2’-FL from d14 to d16 from 6.5 and 12.9% up to 38.2 and 46.6% for both the BF infant and toddler samples. This immediate bifidogenic effect in the lumen of the proximal colon was not observed upon lactose treatment for the BF infant sample, while it was observed, albeit to a milder extent, for the toddler sample with an increase from 10 to 27% in relative abundance. Repeated administration of lactose resulted in more pronounced bifidogenic effects on d26 and d35. Overall, the treatment effects in the lumen of the distal colon and in the mucus of the proximal and distal colon were similar to those observed for the lumen of the proximal colon, although there were minor differences that are likely due to intrinsic variation in the microbial community composition in each environment. Notably, the relative abundance of the *Veillonellaceae* family was reduced after treatment with 2’-FL, but not with lactose, both for the BF infant and toddler. This effect was less pronounced in the mucus compared with the lumen.

#### 3.2.3. Effect of Repeated Administration of 2’-FL versus Lactose on Microbial Activity in the M-SHIME^®^

First, lactose and 2’-FL increased gas production both for the BF infant and toddler sample, with the increase being significantly higher with lactose compared to 2’-FL, consistent with the findings from the short term donor screening (part 1) (Figure S1A). For the BF infant sample, lower gas production for 2’-FL as compared to lactose seemed to be specifically related to lower H_2_ levels (Figure S1B). Also, for the toddler sample a slightly lower H_2_ level was observed upon 2’-FL treatment compared to lactose. For the toddler, a peculiar finding was that repeated administration between d14 and d35 resulted in lower gas production upon exposure to lactose and 2’-FL treatments compared to baseline. This likely resulted from lower H_2_ production for lactose and especially 2’-FL, while CO_2_ levels also decreased for lactose.

**Figure 6:**
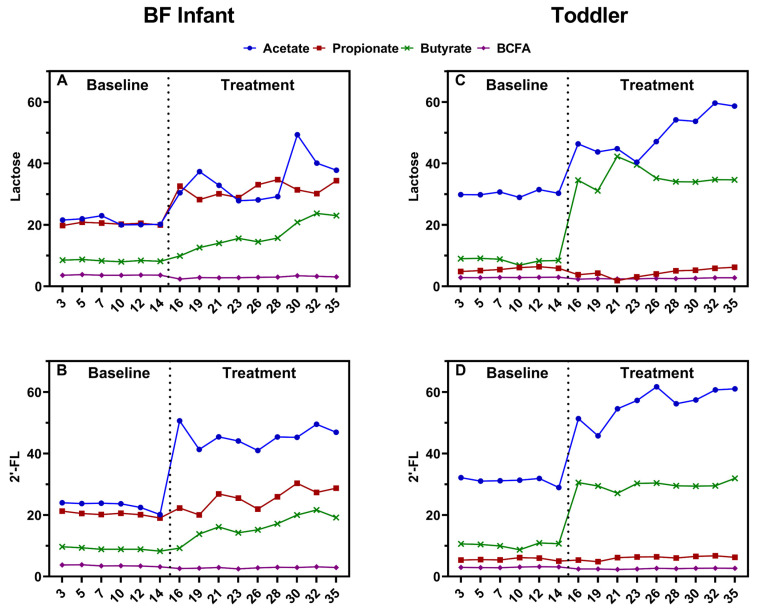
Effect of lactose or 2’-FL on microbial metabolites in the proximal colon of the long-term M-SHIME in vitro system. Time course graphs represent acetate, propionate, butyrate and BCFA levels (mM) in the proximal colon during the baseline (d0–d14) and treatment (d14–d35) periods in the M-SHIME^®^ model: (**A**) lactose treatment to BF infant microbiota; (**B**) 2’-FL treatment to BF infant microbiota; (**C**) lactose treatment to toddler microbiota and (**D**) 2’-FL treatment to toddler microbiota. 2’-FL = 2’fucosyllactose; BCFA = branched-chain fatty acids; BF = breastfed; d = day; M-SHIME^®^ = mucosal simulator of the human intestinal microbial ecosystem.

**Figure 7:**
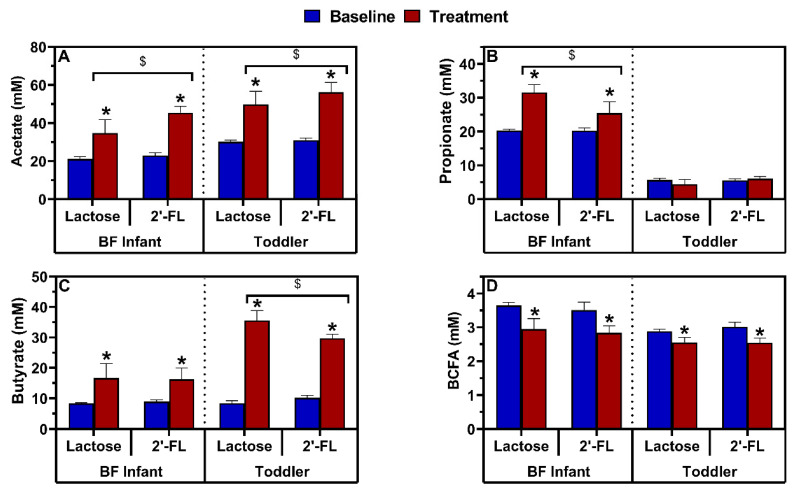
Effect of lactose or 2’-FL on microbial activity in the proximal colon in long-term-term colonic incubations. Bars represent the average (± SD) (**A**) acetate, (**B**) propionate, (**C**) butyrate and (**D**) BCFA levels (mM) at baseline (d0–d14; *n* = 6) and after treatment (d14–d35; *n* = 9) in the simulated proximal colon of the BF infant and toddler M-SHIME^®^. 2’-FL = 2’fucosyllactose; BF = breastfed; d = day; BCFA, branched-chain fatty acids; M-SHIME^®^ = mucosal simulator of the human intestinal microbial ecosystem; SD, standard deviation. For each age group, significant treatment effects (baseline versus treatment) are indicated with an asterisk (*; *p* <0.05), whereas significant treatment effect differences (lactose versus 2’-FL) are indicated with a dollar symbol ($; *p* <0.05), as tested with a two-way ANOVA with Bonferroni correction.

The levels of SCFAs and BCFAs were stable during the baseline period in both the proximal (Figure 6) and distal (Figure S2) colon. In the proximal colon, treatment with both lactose and 2’-FL significantly increased acetate production compared to baseline for both BF infant and toddler microbiota; the increase was greater for 2’-FL when averaged over the entire treatment period (Figure 7A). The time-course data for 2’-FL (Figure 6B,D) highlighted that 2’-FL immediately and strongly increased acetate from the start of the treatment. As acetate is a key metabolite of *Bifidobacteriaceae* members, this correlates with the strong effect of 2’-FL on the relative abundance of this taxonomic group. Propionate levels significantly increased after both lactose and 2’-FL treatment for the BF infant microbiota, with the increase being greater with lactose compared to 2’-FL (Figure 7B). This finding correlates with the higher levels of the propionate producing *Veillonellaceae* family for lactose compared to 2’-FL (Figure 7B). In contrast, the toddler microbiota produced very little propionate at baseline and production did not increase with either lactose or 2’-FL treatment. Butyrate production significantly increased after treatment with either lactose or 2’-FL for both the BF infant and toddler microbiota. For the toddler microbiota, the increase was more pronounced with lactose versus 2’-FL (Figure 7C). In the distal colon, similar treatment effects on acetate (Figure S3A), propionate (Figure S3B), and butyrate (Figure S3C) were observed. Lactate levels were very low in both the proximal and distal colon regardless of the treatment, indicating a good conversion of this intermediate. Finally, compared to baseline, BCFA production was significantly decreased after treatment with either lactose or 2’-FL for both the BF infant and toddler microbiota in the proximal (Figure 7D) and distal (Figure S3D) colon lumen. No differences were observed in alpha diversity indices (Shannon, Chao1), richness or evenness after lactose or 2’-FL treatment (Supplementary Figure S4).

## 4. Discussion

The current study combined two distinct in vitro gut models to address the effect of 2’-FL on the gut microbiome of BF infants and toddlers using lactose as a comparator. First, short-term fecal batch incubations were applied as they allow to more cost-effectively include the aspect of inter-individual variability among donors. This revealed that the BF infant population was characterized by larger inter-individual variations, particularly in terms of 2’-FL fermentation. These data correspond with in vivo findings demonstrating that only some infants have *Bifidobacterium* strains that can use 2’FL [26]. The combined comparison of all read-outs from the first screening by PCA allowed for the selection of a representative donor for each age group for an in-depth study using the M-SHIME^®^ model. This facilitated focus on additional research questions, such as the effect of repeated intake of 2’-FL, the longitudinal effects along the proximal and distal colon and the effects on the luminal and mucosal microbiota. While an M-SHIME^®^ simulation for 3-month old infants was previously developed [47], this is the first report where we demonstrated a stable and diverse microbiota in the toddler M-SHIME^®^ model. The microbial composition of the toddler M-SHIME^®^ after the 14d stabilization period was, like for the BF infant simulation, similar to that of the inoculum in both the lumen and mucus of the proximal and distal colon compartments, with almost all of the families present in the inoculum being represented in the toddler M-SHIME^®^.

In these investigations, 2’-FL fermentation had an immediate and strong bifidogenic effect as demonstrated by the increased relative *Bifidobacteriaceae* abundance from d14 to d16 for both the BF infant and toddler microbiota. Lactose fermentation resulted in a less immediate bifidogenic effect for the toddler microbiota and did not increase *Bifidobacteriaceae* during the first two treatment weeks for the BF infant microbiota. Bifidobacteria are considered beneficial for human health, and low abundance is linked to gastrointestinal and metabolic diseases [66]. A loss of *Bifidobacterium* species during the early colonization of the infant gut can alter the normal progression of the gut microbial community and may negatively impact host health [67]. Therefore, 2’-FL may have a long-term impact on infant and toddler health by potentially increasing the relative abundance of bifidobacteria, and especially their metabolic activity seen in the formation of acetate. Moreover, it is plausible 2’-FL can maintain bifidobacteria presence and activity within the gut microbial community during the diversification of the gut microbiota following the introduction of a complementary diet. Supporting the growth and metabolic activity of *Bifidobacteriaceae* members seems important since many members have been associated with health benefits for the host [68,69].

Additional evidence that the *Bifidobacteriaceae* were a major group of bacteria involved in the fermentation of 2’-FL followed from the observation that acetate, a major metabolite of *Bifidobacteriaceae* [70], was consistently produced at a higher level upon 2’-FL treatment in BF infant and toddler samples in both model systems. These findings are in agreement with a study of the effect of 2’-FL on in vitro colon simulations using infant fecal samples that reported increased acetate production with 2’-FL [71]. Interestingly, acetate has been shown to have immune protective effects both for gastrointestinal pathogens in preclinical in vivo models [72,73]. Hence, 2’FL stimulated the metabolic activity of the microbiota may have important physiologic effects on infant and toddler health.

Another observation that suggests the involvement of *Bifidobacteriaceae* in the fermentation of 2’-FL is linked to the measured gas formation. Fermentation of 2’-FL and lactose resulted in similar acidification. However, only lactose treatment resulted in a strong increase in gas formation. The lower gas production with 2’-FL compared to lactose was consistently observed for BF infants and toddlers in both models. The long-term M-SHIME^®^ model allowed for the conclusion that the observed decrease with 2’-FL was primarily due to H_2_. While fermentation of glycans by colonic microbes usually results in gas production and acidification, fermentation by *Bifidobacteriaceae* results in acidification without gas production, as *Bifidobacteriaceae* do not produce gases [74,75]. This seems to be consistent with in vivo observations for adults, who only experienced mild gas production with 2’-FL as a supplement up to 20 g per day compared to a placebo control [38]. Irrespective of the hypothesis on the involvement of *Bifidobacteriaceae,* the consistently observed mild gas production with 2’-FL is of great interest as it indicates breast milk components like 2’-FL may counterbalance the gas-forming fermentation of other components such as lactose. This may lead to less gut discomfort. Noteworthy, for the toddler M-SHIME^®^ study, we observed that repeated administration of 2’-FL or lactose resulted in lower gas production. This suggests that long-term administration leads to metabolic adaptation and a shift in the ecosystem. To what extent this finding could translate to counterbalance gastrointestinal discomfort symptoms in toddlers deserves further investigation.

Repeated administration of 2’-FL significantly increased production of both butyrate and propionate versus baseline in the BF infant M-SHIME^®^ simulation. For the toddler simulation, there was an immediate and significant increase in butyrate, with no change in propionate production. At the same time, BCFA production was significantly decreased in both the BF infant and toddler simulations. Lactose treatment had similar effects. The increases in butyrate and propionate are noteworthy because both are associated with health benefits. Butyrate is important to colonic health as it is a preferred substrate for colonocytes and is attributed to promoting a normal phenotype and regulating energy metabolism in these cells [76,77]. Butyrate is involved in the regulation of nitrate by colonocytes and limits the bioavailability of luminal oxygen, which together prevent the expansion of potentially pathogenic *Escherichia* and *Salmonella* [78]. Additionally, it is reported to have anti-inflammatory and anti-cancer properties, contributing to increased resistance to enteropathogens and respiratory infections, and playing a role in mitigating weight gain and improving insulin sensitivity [79,80,81]. Propionate has also been shown to have anti-inflammatory and anti-cancer properties, and to reduce lipidogenesis and serum cholesterol, preventing weight gain, improve insulin resistance, protect from hypertensive cardiovascular damage, and to limit the growth of potentially pathogenic bacteria such as *Salmonella* [82]. Additionally, SCFAs may play a role in protecting against food allergies and allergic asthma [83,84,85]. The fact that butyrate and propionate, which can exert important health benefits, were increased with 2’-FL demonstrates that dietary 2’-FL may provide beneficial effects to both infants and toddlers.

With respect to using lactose as a comparator, it has to be noted that in individuals who are able to digest lactose (normolactasia), only a small percentage of lactose reaches the colon because the majority of it is absorbed in the small intestine [86]. HMOs, in contrast, are not absorbed in the upper gastrointestinal tract where only 1–2% are absorbed in infants [87,88]. Given this, even if the benefits of 2’-FL and lactose were equal, one would need an approximately 50× higher dose of lactose than 2’-FL to achieve the same effects. Therefore, 2’-FL has a distinct advantage over lactose in regard to the dose.

## 5. Conclusions

Data obtained using the M-SHIME^®^ model showed a more pronounced bifidogenic effect for both BF infant and toddler gut microbiota with 2’-FL treatment as compared to lactose, which resulted in less gas production and more SCFA production, primarily acetate. The relative abundance of *Bifidobacteriaceae* family increased with both lactose and 2’-FL treatment for the toddler microbiota (albeit more delayed for lactose), but only increased with 2’-FL treatment and not lactose for the BF infant microbiota. Similarly, the screening study showed stimulated metabolic activity most likely related to bifidobacteria. The observations made with the short- and long-term fermentation models of infant and toddler gut microbiota reveal possible roles of 2’-FL mediated through the gut microbiota that may contribute to infant and toddler health and wellbeing.

## Figures and Tables

**Table 1:** Proportional composition at the family level (%) as determined via 16S-targeted Illumina sequencing in the lumen of the proximal colon of the M-SHIME^®^ inoculated with BF infant or toddler fecal samples (before [d14] and after [d16, d26, and d35] treatment with lactose or 2’-FL) (*n* = 1).

Phylum	Family	BF Infant								Toddler							
		Lactose				2’-FL				Lactose				2’-FL			
		d14	d16	d26	d35	d14	d16	d26	d35	d14	d16	d26	d35	d14	d16	d26	d35
*Actinobacteria*	*Atopobiaceae*	-	-	-	-	-	-	-	-	-	-	-	-	-	-	0.03	0.23
	*Bifidobacteriaceae*	5.40	8.24	4.42	17.02	6.55	38.20	38.40	35.21	10.04	27.08	46.15	47.65	12.93	46.60	55.46	57.24
	*Cellulomonadaceae*	-	-	-	-	-	-	-	-	-	-	0.26	0.13	-	-	0.004	0.05
	*Coriobacteriaceae*	0.28	0.08	0.05	0.08	0.18	0.22	0.57	0.26	0.56	0.33	2.83	1.43	1.98	5.43	3.05	8.57
	*Eggerthellaceae*	-	-	-	-	-	-	-	-	-	-	-	0.02	0.01	-	0.004	0.003
	*Microbacteriaceae*	-	-	-	-	-	-	-	-	0.003	0.01	0.02	0.01	-	-	-	0.06
*Bacteroidetes*	*Bacteroidaceae*	1.39	6.74	0.56	1.15	2.35	1.45	1.37	0.84	15.13	4.07	11.74	13.14	21.16	4.55	10.63	14.50
	*Marinifilaceae*	-	-	-	-	-	-	-	-	-	-	-	-	-	-	-	-
	*Porphyromonadaceae*	0.09	0.35	0.24	0.31	0.29	0.16	0.23	0.24	-	-	-	-	-	-	-	-
	*Rikenellaceae*	0.06	0.23	0.75	0.35	0.09	0.07	0.17	0.46	0.003	0.02	0.02	0.01	0.01	0.01	0.01	-
	*Tannerellaceae*	-	-	-	-	-	-	-	-	0.29	0.09	0.21	0.54	0.53	0.03	0.30	0.14
*Firmicutes*	*Acidaminococcaceae*	-	-	-	-	-	-	-	-	0.07	0.14	2.27	1.56	0.37	0.21	1.50	1.20
	*Clostridiaceae_1*	0.01	0.02	-	-	0.01	0.01	-	-	-	0.02	-	-	-	-	-	-
	*Clostridiales Incertae_Sedis_XI*	-	0.12	-	-	0.005	0.01	-	-	-	-	-	-	-	-	-	-
	*Clostridiales unclassified*	-	-	-	-	-	-	-	-	-	-	-	-	-	-	-	-
	*Erysipelotrichaceae*	-	-	-	-	-	-	-	-	0.03	0.01	-	0.04	0.02	0.01	0.02	0.02
	*Eubacteriaceae*	-	-	-	-	-	-	-	-	0.01	-	0.00	0.04	0.02	0.01	-	0.01
	*Lachnospiraceae*	4.32	14.97	27.30	9.97	4.40	26.59	7.67	10.99	49.78	24.91	5.12	11.37	36.15	14.58	10.51	4.47
	*Lactobacillaceae*	-	-	-	-	-	-	-	-	-	-	0.01	0.01	-	-	-	-
	*Ruminococcaceae*	0.39	1.34	0.30	0.60	0.85	0.20	0.09	0.17	0.10	0.04	0.01	9.67	0.04	-	0.04	0.37
	*Streptococcaceae*	-	-	-	-	-	-	-	-	-	-	-	-	-	-	-	-
	*Veillonellaceae*	87.81	67.41	65.99	69.76	84.71	32.66	50.62	51.33	22.00	42.32	30.33	12.32	24.76	27.61	17.00	12.20
*Proteobacteria*	*Burkholderiaceae*	-	-	-	-	-	-	-	-	0.22	0.04	0.16	0.33	0.08	-	0.12	0.34
	*Campylobacteraceae*	-	-	-	-	-	-	-	-	-	-	-	-	-	-	-	-
	*Desulfovibrionaceae*	-	-	-	-	-	-	-	-	0.16	0.14	0.02	0.02	0.11	0.03	0.07	0.02
	*Enterobacteriaceae*	0.03	0.01	0.01	0.26	0.05	0.01	0.14	0.09	1.31	0.64	0.71	1.18	1.63	0.84	1.14	0.53
	*Pseudomonadaceae*	0.06	0.11	0.24	0.29	0.09	0.08	0.41	0.15	0.16	0.11	0.11	0.07	0.03	0.01	0.08	0.04
	*Sutterellaceae*	0.06	0.10	0.07	0.14	0.20	0.09	0.25	0.18	-	-	-	-	-	-	-	-
	*Xanthomonadaceae*	0.02	0.04	0.03	-	0.05	0.06	0.03	0.01	0.11	0.003	0.01	0.40	0.08	0.01	0.01	0.01
*Verrucomicrobia*	*Akkermansiaceae*	-	-	-	-	-	-	-	-	-	-	-	0.01	-	-	-	-

M-SHIME^®^ = mucosal simulator of the human intestinal microbial ecosystem; BF = breastfed; d = day; 2’-FL = 2’fucosyllactose.

## Data Availability

Derived data supporting the findings of this study are available at NCBI repository with the following access number PRJNA701368 or directly from the corresponding author M.M. upon request.

## Supplementary Materials

The following are available online at https://www.mdpi.com/2072-6643/13/3/726/s1, Table S1: Proportional microbial composition at the family level (%) as determined via 16S-targeted Illumina sequencing; Table S2: Proportional microbial composition at the family level (%) as determined via 16S-targeted Illumina sequencing in the lumen of the distal colon of the M-SHIME^®^ inoculated with BF infant or toddler fecal samples before (d14) and after (d16, d26, and d35) treatment with lactose or 2’-FL (*n* = 1); Table S3: Proportional microbial composition at the family level (%) as determined via 16S-targeted Illumina sequencing in the mucus of the proximal colon of the M-SHIME^®^ inoculated with BF infant or toddler fecal samples before (d14) and after (d16, d26, and d35) treatment with lactose or 2’-FL (*n* = 1); Table S4: Proportional microbial composition at the family level (%) as determined via 16S-targeted Illumina sequencing in the mucus of the distal colon of the M-SHIME^®^ inoculated with BF infant or toddler fecal samples before (d14) and after (d16, d26, and d35) treatment with lactose or 2’-FL (*n* = 1); Figure S1: Gas production and composition; Figure S2: Acetate, propionate, butyrate, and BCFA levels (mM) in the distal colon during the baseline (d0–d14) and treatment periods (d14–d35) periods in the M-SHIME^®^ model; Figure S3: Average (± SD) (A) acetate, (B) propionate, (C) butyrate, and (D) BCFA levels (mM) at baseline (d0–d14; *n* = 6) and after treatment (d14–d35; *n* = 9) in the simulated distal colon of the BF infant and toddler M-SHIME^®^. Figure S4. Alpha diversity indices (Chao1 and Shannon), evenness and richness at family level for luminal (A) and mucosal (B) compartments.
