# Supplementary material for: A Comparison of the In Vitro Effects of 2’Fucosyllactose and Lactose on the Composition and Activity of Gut Microbiota from Infants and Toddlers
Source: Nutrients. 2021 Feb 25;13(3):726. doi: 10.3390/nu13030726 (PMC7996240; doi:10.3390/nu13030726)
Supplement: Supplementary file 1 [file nutrients-13-00726-s001.pdf]

nutrients MDPI

Supplementary Material

1. Supplementary Tables

Supplementary Table S1. Proportional microbial composition at the family level (%) as determined via 16S-targeted Illumina sequencing.

Phylum	Family	BF Infant					Toddler				
		Lumen		Mucus		Inoculum	Lumen		Mucus		Inoculum
		PC	DC	PC	DC		PC	DC	PC	DC	
Actinobacteria	*Atopobiaceae*	-	-	-	-	-	0.03	0.04	0.04	0.01	-
	Bifidobacteriaceae	19.18	17.97	30.47	28.05	98.26	37.89	33.63	48.46	44.64	30.03
	Cellulomonadaceae	-	-	-	-	-	0.06	0.05	0.09	0.05	-
	Coriobacteriaceae	0.21	0.39	0.35	0.41	0.01	3.02	3.24	3.53	3.43	0.40
	Eggerthellaceae	-	-	-	-	-	0.00	0.02	0.05	0.17	0.45
	Microbacteriaceae	-	-	-	-	-	0.01	0.03	0.01	0.01	-
Bacteroidetes	*Bacteroidaceae*	1.98	0.80	1.08	0.64	0.002	11.87	12.31	3.64	5.11	16.23
	Marinifilaceae	-	-	-	-	-	-	-	-	-	0.11
	Porphyromonadaceae	0.24	0.19	**0.37**	**0.20**	0.001	-	-	-	-	-
	Rikenellaceae	0.27	0.13	0.47	0.32	-	**0.01**	**0.05**	0.03	0.05	0.62
	Tannerellaceae	-	-	-	-	-	**0.27**	**0.62**	0.36	0.46	6.39
Firmicutes	*Acidaminococcaceae*	-	-	-	-	-	0.91	1.33	3.23	2.81	0.002
	Clostridiaceae_1	0.01	0.004	0.41	0.15	-	0.00	0.00	**0.03**	**0.80**	-
	Clostridiales_Incertae_Sedis_XI	0.02	0.28	**1.54**	**10.70**	0.001	-	-	-	-	-
	Clostridiales_unclassified	-	-	-	-	-	-	0.02	0.03	0.19	-
	Erysipelotrichaceae	-	-	-	-	-	**0.02**	**0.14**	0.10	2.04	0.28
	Eubacteriaceae	-	-	-	-	-	0.01	0.05	**0.18**	**0.98**	0.02
	Lachnospiraceae	**13.28**	**6.06**	21.19	16.71	0.52	**19.61**	**25.50**	16.75	19.02	21.56
	Lactobacillaceae	-	-	-	-	-	0.00	0.01	0.01	0.25	-
	Ruminococcaceae	0.49	0.64	1.30	1.03	0.003	**1.28**	**2.62**	**0.33**	**1.89**	20.80
	Streptococcaceae	-	-	-	-	-	-	-	-	-	0.12
	Veillonellaceae	63.78	73.19	42.36	41.42	0.69	23.57	18.57	22.07	17.15	0.28
Proteobacteria	*Burkholderiaceae*	-	-	-	-	-	0.16	0.23	0.13	0.32	-
	Campylobacteraceae	-	-	-	-	-	-	-	-	0.02	-
	Desulfovibrionaceae	-	-	-	-	-	0.07	0.08	0.24	0.26	0.05
	Enterobacteriaceae	**0.08**	**0.01**	0.02	0.03	0.27	1.00	0.59	0.65	0.26	0.38
	Pseudomonadaceae	0.18	0.14	0.01	0.03	-	**0.08**	**0.59**	0.01	0.01	-
	Sutterellaceae	0.14	0.16	0.28	0.20	-	-	-	-	-	-
	Xanthomonadaceae	**0.03**	**0.01**	0.01	0.01	0.001	0.08	0.06	0.01	0.01	-
Verrucomicrobia	*Akkermansiaceae*	-	-	-	-	-	0.001	0.17	0.003	0.003	2.16

Data were averaged along the entire experiment (days 14, 16, 26, and 35) in the lumen and mucus of the proximal and distal colon of the M-SHIME® (n = 8 for each environment), both for BF infant and toddler simulations, together with the composition of the inocula (n = 3) used to seed the *in vitro* models. Significant differences

in relative abundance between the proximal and distal colon are indicated in bold, and between the luminal and mucosal compartments are indicated by underlining, as tested by a paired two-sided Student's t-test ($p < 0.05$).
BF = breastfed; PC = proximal colon; DC = distal colon; M-SHIME® = mucosal simulator of the human intestinal microbial ecosystem

Supplementary Table S2. Proportional microbial composition at the family level (%) as determined via 16S-targeted Illumina sequencing in the lumen of the distal colon of the M-SHIME® inoculated with BF infant or toddler fecal samples before (d14) and after (d16, d26, and d35) treatment with lactose or 2′-FL (n = 1).

Phylum	Family	BF infant								Toddler							
		Lactose				2′-FL				Lactose				2′-FL			
		d14	d16	d26	d35	d14	d16	d26	d35	d14	d16	d26	d35	d14	d16	d26	d35
Actinobacteria	*Atopobiaceae*	-	-	-	-	-	-	-	-	-	-	-	-	-	-	0.03	0.29
	Bifidobacteriaceae	3.61	2.78	0.62	12.55	4.42	31.77	45.80	42.17	6.11	32.50	45.48	34.25	11.44	46.30	50.83	42.15
	Cellulomonadaceae	-	-	-	-	-	-	-	-	0.004	0.003	0.14	0.17	-	0.00	0.00	0.04
	Coriobacteriaceae	0.32	0.10	0.02	0.10	0.29	0.09	0.73	1.46	0.57	0.57	5.19	3.05	1.26	3.54	2.11	9.60
	Eggerthellaceae	-	-	-	-	-	-	-	-	0.06	0.04	0.01	0.02	0.03	0.01	0.01	0.01
	Microbacteriaceae	-	-	-	-	-	-	-	-	0.004	0.003	0.04	0.02	-	0.01	0.004	0.15
Bacteroidetes	*Bacteroidaceae*	1.54	0.99	0.20	0.53	1.66	0.60	0.42	0.43	20.02	8.81	12.39	13.82	14.02	6.01	10.25	13.12
	Marinifilaceae	-	-	-	-	-	-	-	-	-	-	-	-	-	-	-	-
	Porphyromonadaceae	0.17	0.09	0.13	0.29	0.28	0.11	0.24	0.20	-	-	-	-	-	-	-	-
	Rikenellaceae	0.06	0.11	0.08	0.38	-	0.05	0.13	0.22	0.06	0.09	0.02	0.09	0.03	0.01	0.02	0.06
	Tannerellaceae	-	-	-	-	-	-	-	-	0.94	0.56	0.26	0.35	1.09	0.21	0.69	0.83
Firmicutes	*Acidaminococcaceae*	-	-	-	-	-	-	-	-	0.08	0.11	2.26	1.83	0.30	0.42	3.76	1.90
	Clostridiaceae_1	0.01	0.01	-	-	0.01	-	-	-	-	-	-	-	-	0.01	-	-
	Clostridiales Incertae_Sedis_XI	1.22	0.32	0.08	0.14	0.34	0.05	0.03	0.07	-	-	-	-	-	-	-	-
	Clostridiales unclassified	-	-	-	-	-	-	-	-	0.02	0.003	-	0.01	-	0.003	0.02	0.12
	Erysipelotrichaceae	-	-	-	-	-	-	-	-	0.20	0.23	0.05	0.11	0.05	0.04	0.24	0.17
	Eubacteriaceae	-	-	-	-	-	-	-	-	0.02	0.01	0.02	-	0.02	0.05	0.14	0.15
	Lachnospiraceae	6.93	4.61	2.97	4.14	5.36	16.80	3.71	3.95	42.51	30.65	9.74	17.44	49.73	24.66	17.78	11.49
	Lactobacillaceae	-	-	-	-	-	-	-	-	-	-	0.02	0.03	-	0.003	-	-
	Ruminococcaceae	1.14	1.00	0.59	0.77	0.89	0.25	0.17	0.28	0.73	0.74	1.35	13.92	0.70	0.38	0.16	2.99
	Streptococcaceae	-	-	-	-	-	-	-	-	-	-	-	-	-	-	-	-
	Veillonellaceae	84.51	89.71	94.97	80.75	86.44	50.12	48.10	50.91	27.05	24.88	21.66	11.42	20.27	17.89	11.29	14.11
Proteobacteria	*Burkholderiaceae*	-	-	-	-	-	-	-	-	0.08	0.09	0.27	0.57	0.02	0.05	0.53	0.28
	Campylobacteraceae	-	-	-	-	-	-	-	-	-	-	-	-	-	-	-	-
	Desulfovibrionaceae	-	-	-	-	-	-	-	-	0.16	0.16	0.02	0.04	0.09	0.03	0.04	0.10
	Enterobacteriaceae	0.01	0.01	0.01	0.06	-	-	-	0.02	0.42	0.22	0.42	1.02	0.36	0.21	1.20	0.87
	Pseudomonadaceae	0.11	0.11	0.26	0.16	0.03	0.01	0.34	0.11	0.74	0.26	0.24	0.69	0.49	0.13	0.80	1.35
	Sutterellaceae	0.30	0.11	0.05	0.11	0.19	0.11	0.25	0.16	-	-	-	-	-	-	-	-
	Xanthomonadaceae	0.02	0.01	0.01	-	0.03	0.01	0.02	-	0.12	0.02	0.02	0.29	0.01	-	0.02	0.02
Verrucomicrobia	*Akkermansiaceae*	-	-	-	-	-	-	-	-	0.02	0.03	0.36	0.78	-	-	0.05	0.15

2′-FL = 2′fucosyllactose; BF = breastfed; d = day; M-SHIME® = mucosal simulator of the human intestinal microbial ecosystem.

Supplementary Table S3. Proportional microbial composition at the family level (%) as determined via 16S-targeted Illumina sequencing in the mucus of the proximal colon of the M-SHIME® inoculated with BF infant or toddler fecal samples before (d14) and after (d16, d26, and d35) treatment with lactose or 2′-FL (n = 1).

**Phylum**	**Family**	**BF infant**								**Toddler**							
		**Lactose**				**2′-FL**				**Lactose**				**2′-FL**			
		**d14**	**d16**	**d26**	**d35**	**d14**	**d16**	**d26**	**d35**	**d14**	**d16**	**d26**	**d35**	**d14**	**d16**	**d26**	**d35**
Actinobacteria	*Atopobiaceae*	-	-	-	-	-	-	-	-	0.27	-	-	-	-	-	-	0.01
	Bifidobacteriaceae	16.47	14.14	17.34	61.66	16.04	55.69	51.51	10.92	63.84	21.70	44.49	55.68	60.29	26.67	36.19	57.36
	Cellulomonadaceae	-	-	-	-	-	-	-	-	0.06	0.00	0.05	0.11	0.23	-	-	0.01
	Coriobacteriaceae	0.30	0.23	0.18	0.10	0.56	0.53	0.69	0.18	6.80	3.79	3.20	1.53	0.63	3.18	1.81	4.92
	Eggerthellaceae	-	-	-	-	-	-	-	-	0.01	0.73	0.14	0.03	0.02	0.31	0.05	0.07
	Microbacteriaceae	-	-	-	-	-	-	-	-	0.01	-	-	-	0.01	-	-	0.00
Bacteroidetes	*Bacteroidaceae*	0.96	1.26	0.43	2.17	1.67	0.92	0.51	0.74	1.80	8.06	4.05	7.71	5.41	4.59	5.68	2.60
	Marinifilaceae	-	-	-	-	-	-	-	-	-	-	-	-	-	-	-	-
	Porphyromonadaceae	0.19	0.27	0.34	0.74	0.36	0.18	0.25	0.61	-	-	-	-	-	-	-	-
	Rikenellaceae	0.13	0.14	1.09	0.22	0.27	0.09	0.67	1.13	0.00	0.08	0.05	0.05	0.05	0.02	0.02	0.03
	Tannerellaceae	-	-	-	-	-	-	-	-	0.16	0.76	0.27	0.41	0.25	0.62	0.44	0.29
Firmicutes	*Acidaminococcaceae*	-	-	-	-	-	-	-	-	3.51	0.22	10.52	2.71	2.00	0.50	0.21	1.47
	Clostridiaceae_1	1.20	0.34	0.03	0.03	1.13	0.50	-	0.03	-	1.19	0.48	0.18	0.01	1.02	1.69	1.36
	Clostridiales Incertae_Sedis_XI	0.37	0.06	0.22	2.94	1.33	0.32	0.03	7.07	-	-	-	-	-	-	-	-
	Clostridiales unclassified	-	-	-	-	-	-	-	-	0.00	0.24	0.07	0.18	-	0.13	0.07	0.28
	Erysipelotrichaceae	-	-	-	-	-	-	-	-	0.02	1.76	0.65	0.32	0.03	2.72	7.63	1.69
	Eubacteriaceae	-	-	-	-	-	-	-	-	0.32	1.12	0.89	0.44	0.12	1.10	1.22	1.90
	Lachnospiraceae	18.08	26.34	40.23	13.35	16.93	28.99	16.31	9.25	4.41	33.17	17.31	11.67	12.30	25.83	25.52	15.67
	Lactobacillaceae	-	-	-	-	-	-	-	-	-	-	-	0.01	-	1.97	-	-
	Ruminococcaceae	1.96	1.63	0.76	0.57	1.96	1.79	0.91	0.77	0.47	2.15	1.36	1.59	0.18	4.17	3.16	1.40
	Streptococcaceae	-	-	-	-	-	-	-	-	-	-	-	-	-	-	-	-
	Veillonellaceae	59.87	55.23	39.03	17.91	58.89	10.33	28.61	69.02	17.88	22.80	16.03	16.93	18.10	25.35	15.36	10.35
Proteobacteria	*Burkholderiaceae*	-	-	-	-	-	-	-	-	0.23	0.59	0.14	0.21	0.24	0.57	0.21	0.25
	Campylobacteraceae	-	-	-	-	-	-	-	-	-	0.18	-	-	-	0.02	-	-
	Desulfovibrionaceae	-	-	-	-	-	-	-	-	0.09	0.83	0.09	0.07	0.03	0.49	0.33	0.07
	Enterobacteriaceae	0.04	-	0.01	0.01	0.09	0.03	0.02	0.01	0.10	0.43	0.09	0.11	0.11	0.69	0.39	0.18
	Pseudomonadaceae	0.02	-	-	-	0.07	-	0.01	0.01	0.00	0.05	-	-	-	0.02	-	0.03
	Sutterellaceae	0.37	0.18	0.24	0.16	0.33	0.41	0.34	0.21	-	-	-	-	-	-	-	-
	Xanthomonadaceae	-	-	0.01	0.01	0.04	0.01	0.02	0.01	0.00	0.05	0.00	-	0.01	0.03	0.00	-
Verrucomicrobia	*Akkermansiaceae*	-	-	-	-	-	-	-	-	-	-	-	0.01	-	-	-	-

2′-FL = 2′fucosyllactose; BF = breastfed; d = day; M-SHIME® = mucosal simulator of the human intestinal microbial ecosystem.

Supplementary Table S4. Proportional microbial composition at the family level (%) as determined via 16S-targeted Illumina sequencing in the mucus of the distal colon of the M-SHIME® inoculated with BF infant or toddler fecal samples before (d14) and after (d16, d26, and d35) treatment with lactose or 2′-FL (n = 1).

**Phylum**	**Family**	**BF infant**								**Toddler**							
		**Lactose**				**2′-FL**				**Lactose**				**2′-FL**			
		**d14**	**d16**	**d26**	**d35**	**d14**	**d16**	**d26**	**d35**	**d14**	**d16**	**d26**	**d35**	**d14**	**d16**	**d26**	**d35**
Actinobacteria	*Atopobiaceae*	-	-	-	-	-	-	-	-	-	-	-	-	-	-	0.01	0.08
	Bifidobacteriaceae	13.53	9.86	10.81	26.88	40.36	20.69	67.77	34.52	21.70	44.49	55.68	60.29	26.67	36.19	57.36	54.76
	Cellulomonadaceae	-	-	-	-	-	-	-	-	0.00	0.05	0.11	0.23	-	-	0.01	0.04
	Coriobacteriaceae	0.13	0.18	0.09	0.18	1.27	0.17	0.65	0.62	3.79	3.20	1.53	0.63	3.18	1.81	4.92	8.39
	Eggerthellaceae	-	-	-	-	-	-	-	-	0.73	0.14	0.03	0.02	0.31	0.05	0.07	0.04
	Microbacteriaceae	-	-	-	-	-	-	-	-	-	-	-	0.01	-	-	0.00	0.04
Bacteroidetes	*Bacteroidaceae*	1.01	1.14	0.18	0.70	0.47	0.65	0.34	0.64	8.06	4.05	7.71	5.41	4.59	5.68	2.60	2.79
	Marinifilaceae	-	-	-	-	-	-	-	-	-	-	-	-	-	-	-	-
	Porphyromonadaceae	0.26	0.20	0.17	0.37	0.17	0.17	0.11	0.18	-	-	-	-	-	-	-	-
	Rikenellaceae	0.06	0.23	0.55	0.64	0.15	0.08	0.30	0.53	0.08	0.05	0.05	0.05	0.02	0.02	0.03	0.09
	Tannerellaceae	-	-	-	-	-	-	-	-	0.76	0.27	0.41	0.25	0.62	0.44	0.29	0.67
Firmicutes	*Acidaminococcaceae*	-	-	-	-	-	-	-	-	0.22	10.52	2.71	2.00	0.50	0.21	1.47	4.86
	Clostridiaceae_1	0.43	0.28	-	0.07	0.05	0.30	0.02	0.08	1.19	0.48	0.18	0.01	1.02	1.69	1.36	0.46
	Clostridiales Incertae_Sedis_XI	24.98	15.42	5.15	0.33	2.10	14.03	0.12	23.43	-	-	-	-	-	-	-	-
	Clostridiales unclassified	-	-	-	-	-	-	-	-	0.24	0.07	0.18	-	0.13	0.07	0.28	0.55
	Erysipelotrichaceae	-	-	-	-	-	-	-	-	1.76	0.65	0.32	0.03	2.72	7.63	1.69	1.54
	Eubacteriaceae	-	-	-	-	-	-	-	-	1.12	0.89	0.44	0.12	1.10	1.22	1.90	1.05
	Lachnospiraceae	15.99	12.96	18.80	29.50	6.13	19.27	13.02	18.03	33.17	17.31	11.67	12.30	25.83	25.52	15.67	10.66
	Lactobacillaceae	-	-	-	-	-	-	-	-	-	-	0.01	-	1.97	-	-	-
	Ruminococcaceae	1.67	1.90	0.52	0.70	0.46	1.68	0.45	0.88	2.15	1.36	1.59	0.18	4.17	3.16	1.40	1.10
	Streptococcaceae	-	-	-	-	-	-	-	-	-	-	-	-	-	-	-	-
	Veillonellaceae	41.46	57.39	63.61	40.29	48.32	42.68	16.94	20.68	22.80	16.03	16.93	18.10	25.35	15.36	10.35	12.31
Proteobacteria	*Burkholderiaceae*	-	-	-	-	-	-	-	-	0.59	0.14	0.21	0.24	0.57	0.21	0.25	0.35
	Campylobacteraceae	-	-	-	-	-	-	-	-	0.18	-	-	-	0.02	-	-	-
	Desulfovibrionaceae	-	-	-	-	-	-	-	-	0.83	0.09	0.07	0.03	0.49	0.33	0.07	0.14
	Enterobacteriaceae	0.02	0.05	0.01	0.07	0.11	-	-	0.02	0.43	0.09	0.11	0.11	0.69	0.39	0.18	0.06
	Pseudomonadaceae	0.02	-	-	0.03	0.18	-	-	-	0.05	-	-	-	0.02	-	0.03	-
	Sutterellaceae	0.31	0.20	0.07	0.18	0.20	0.13	0.22	0.27	-	-	-	-	-	-	-	-
	Xanthomonadaceae	-	0.03	0.02	0.01	-	-	-	-	0.05	0.00	-	0.01	0.03	0.00	-	-
Verrucomicrobia	*Akkermansiaceae*	-	-	-	-	-	-	-	-	-	-	0.01	-	-	-	-	0.01

2′-FL = 2′fucosyllactose; BF = breastfed; d = day; M-SHIME® = mucosal simulator of the human intestinal microbial ecosystem.

2. Supplementary Figures

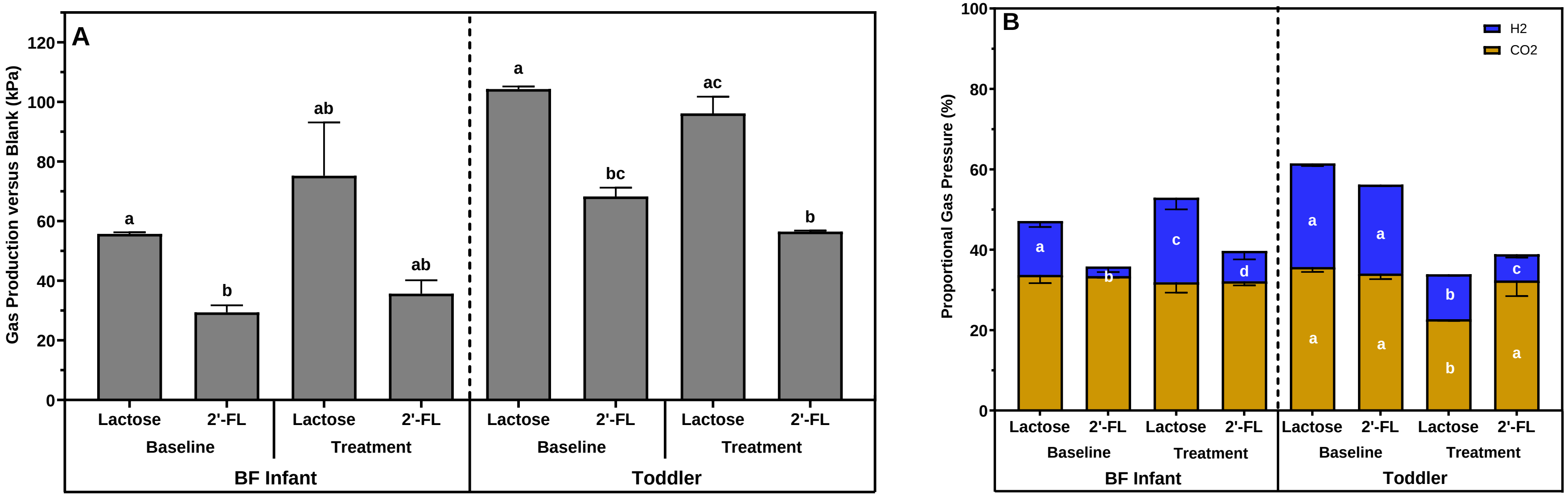

Supplementary Figure S1. Gas production and composition. Assessed in a standardized offline setup that involved collecting samples from the proximal colon of the M-SHIME® and subsequently treating them with lactose or 2′-FL in closed reactors during 48h. This offline gas assessment was performed on samples collected before (d14 - baseline) and after treatment (d35): (A) gas production versus the blank (kPa; 0–48h) and (B) gas composition (mol%; 48h). 2′-FL = 2′fucosyllactose; BF = breastfed; M-SHIME® = mucosal simulator of the human intestinal microbial ecosystem. For each age group, significant differences (A) between the different conditions and (B) between the different conditions for a given gas, are indicated with different letters (a, b, c, d; $p < 0.05$), as tested with a two-way ANOVA with Bonferroni correction.

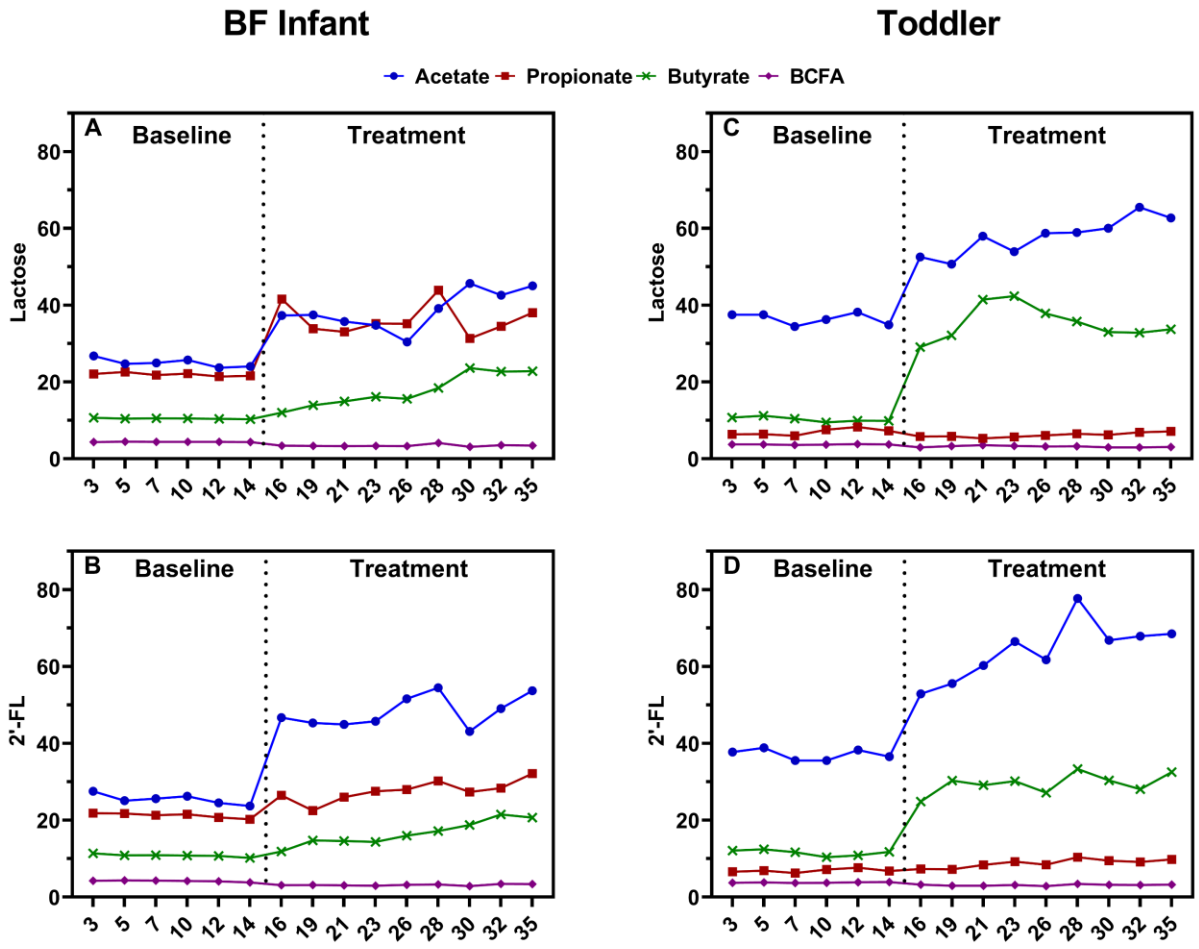

Supplementary Figure S2. Acetate, propionate, butyrate, and BCFA levels (mM) in the distal colon during the baseline (d0–d14) and treatment (d14–d35) periods in the M-SHIME® model: (A) lactose treatment to BF infant microbiota; (B) 2′-FL treatment to BF infant microbiota; (C) lactose treatment to toddler microbiota; (D) 2′-FL treatment to toddler microbiota. 2′-FL = 2′fucosyllactose; BCFA = branched-chain fatty acids; BF = breastfed; d = day; M-SHIME® = mucosal simulator of the human intestinal microbial ecosystem.

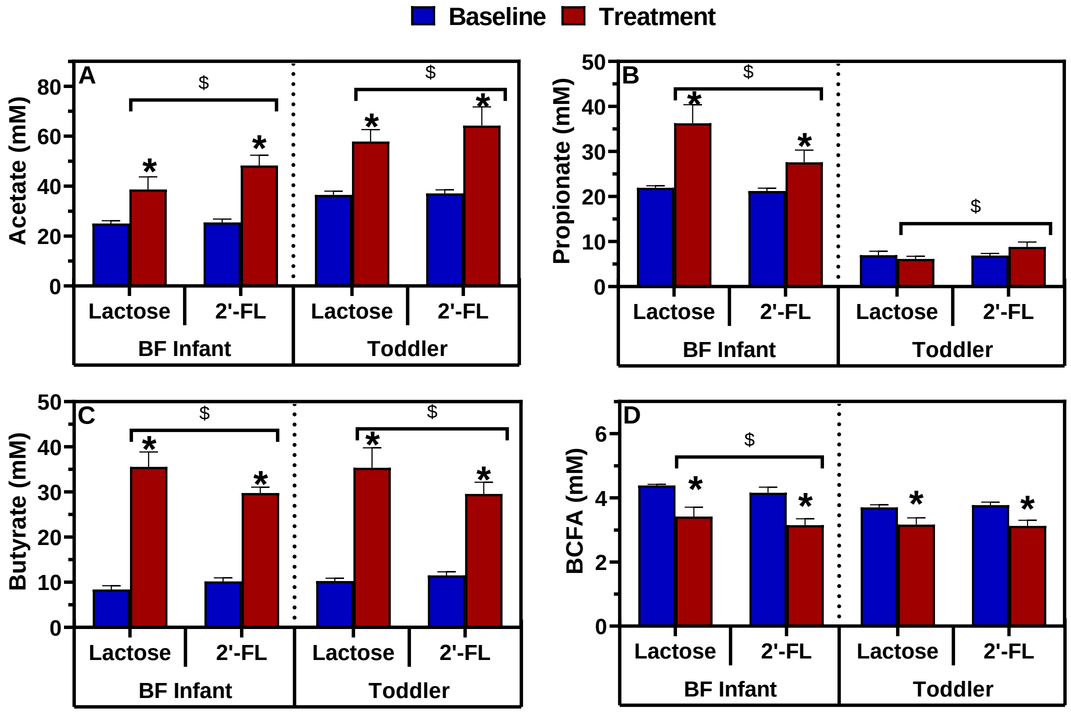

Supplementary Figure S3. Average (± SD) (A) acetate, (B) propionate, (C) butyrate and (D) BCFA levels (mM) at baseline (d0–d14; n = 6) and after treatment (d14–d35; n = 9) in the simulated distal colon of the BF infant and toddler M-SHIME®. 2′-FL = 2′fucosyllactose; BF = breastfed; BCFA = branched-chain fatty acids; M-SHIME® = mucosal simulator of the human intestinal microbial ecosystem; SD = standard deviation. For each age group, significant treatment effects (baseline versus treatment) are indicated with an asterisk (*; p < 0.05), whereas significant treatment effect differences (lactose versus 2′-FL) are indicated with a dollar symbol ($; p < 0.05), as tested with a two-way ANOVA with Bonferroni correction.

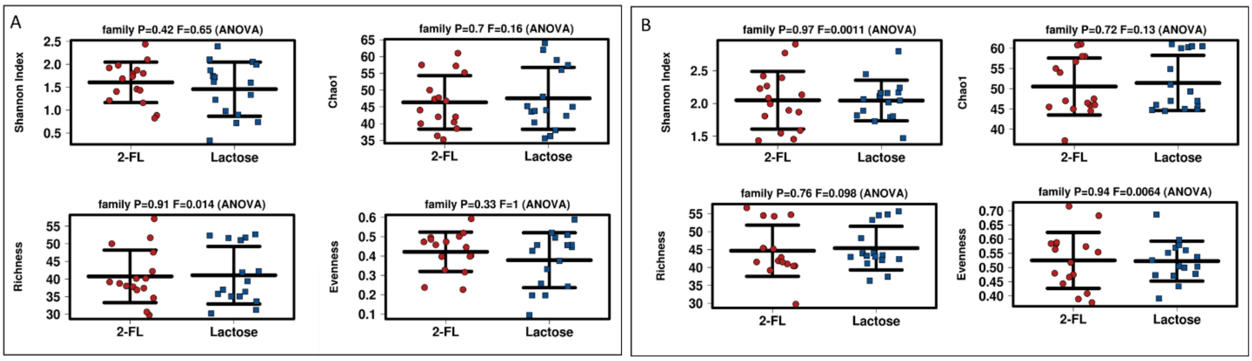

Supplementary Figure S4. Alpha diversity indices (Chao1 and Shannon), evenness and richness at family level for luminal (A) and mucosal (B) compartments.

Diversity indices, evenness and richness were also calculated in the proximal and distal colon compartments, without significant differences observed (data not shown).
